# Relational POI recommendation model combined with geographic information

**DOI:** 10.1371/journal.pone.0266340

**Published:** 2022-04-15

**Authors:** Ke Li, Haitao Wei, Xiaohui He, Zhihui Tian

**Affiliations:** 1 The School of the Geo-Science & Technology, Zhengzhou University, Zhengzhou, Henan, China; 2 Joint Laboratory of Eco-Meteorology, Zhengzhou University, Chinese Academy of Meteorological Sciences, Zhengzhou, Henan, China; GC Women University Sialkot, PAKISTAN

## Abstract

Point of interest (POI) recommendation is a popular personalized location-based service. This paper proposes a Geographic Personal Matrix Factorization (GPMF) model that makes effective use of geographic information from the perspective of the relationship between POIs and users. This model considers the role of geographic information from multiple perspectives based on the locational relationship among users, the distributional relationship between users and POIs, and the proximity and clustering relationship among POIs. The GPMF mines the influence of geographic information on different objects and carries out unique modeling through cosine similarity, non-linear function, and k nearest neighbor (KNN). This study explored the influence of geographic information on POI recommendation through extensive experiments with data from Foursquare. The result shows that GPMF performs better than the commonly used POI recommendation algorithm in terms of both precision and recall. Geographic information through proximity relations effectively improves the recommendation algorithm.

## 1. Introduction

With the rapid development of wireless communication technology and mobile Internet, people can quickly and easily obtain their location through mobile devices, and share their location information with other users through location-based social networks (LBSN) [1]. Some examples of commonly used LBSNs are Foursquare, Gowalla, and Yelp [2]. Due to the wide application of LBSN and other location-based services, user preference mining and point of interest (POI) recommendations have become common. Users can display check-in records and share experiences on LBSN. These records include real-time location, access time, ratings, comments, and other information [3]. There are many users and POIs on LBSN, and they generate massive amounts of information. Information overload is a challenge and dilemma that must be addressed for POI recommendations. Therefore, processing and utilizing this information is key to perfecting POI recommendations. The task of POI recommendation is to mine the user’s preferences or interests through the user’s historical check-in records on LBSN and recommend places that the user has not previously visited but may be interested in. This task has important practical significance and high theoretical value [4].

There are many factors that affect POI recommendation, such as geographic factors(distance, distribution, proximity, etc.), time influence, popularity, and review information [5]. It is very difficult to process and analyze human behavior on a large scale and in a wide range. Just like the check-in records used in POI recommendations, the information contained in a check-in record is very limited, but the factors that affect a check-in are highly complex. There are many unknown human factors that drive a user to check-in, but there is no clear record of this information. Nevertheless, although we cannot know how many personal factors affect a given check-in, we can explicate the crucial role of users in POI recommendation by analyzing user relationships or group behavior patterns. In addition, numerous studies have confirmed the important role of geographic information in POI recommendations [6–9]. Although there are differences in the methods of using and quantifying (such as common power law formulas, kernel density formulas, and Gaussian kernel functions), they point to one basic rule: The closer the distance, the stronger the effect, and the farther the distance, the weaker the effect.

Many previous studies have investigated the influence of geographic factors, but most of them obtained a check-in probability or transition probability affected by geographic factors [10–12]; they did not analyze the influence of geographic factors from multiple perspectives. The method proposed in this paper first analyzes the structure of POI recommendation and then considers the role of geographic factors between users, between users and POI, and between POIs. Moreover, we considered the influence of geographic factors from the perspective of the relationships between different individuals. Our method can be summarized as follows. (1) We observed the locational relationship of center of user activity range, calculated the geographically similar users, and used cosine similarity to calculate the similarity between users. This step can add the geographic influence among users. (2) We calculated the life circle of each user based on the coordinates of historical check-in information, and determined whether a POI is in the user’s life circle. This step can connect the relationship between users and POIs, and add the geographic influence between users and POIs. (3) We analyzed the proximity and cluster relationships between POIs, calculated the geographic neighbors of POIs by k nearest neighbor (KNN), used the aggregation information of POIs to calculated the cluster to which the POIs belong and the role of POI clusters. This step can add the geographic influence among POIs. Finally, we added the contend of the above calculation to the matrix factorization model.

This study establishes a Geographic Personal Matrix Factorization model (GPMF), which can consider geographic information through multiple perspectives. The organization of the paper is as follows. Section 2 summarizes the related work. Section 3 provides an overview of the GPMF. Section 4 presents the results of an experimental evaluation of the proposed method. Section 5 concludes this paper.

## 2. Related work

### 2.1 POI recommendation

POI recommendation made a late appearance in the field of recommendation systems [13]. Its rise was mainly due to the development of LBSN, and it rapidly became common in our lives [14]. Compared to other types of recommendation (such as movie recommendation, music recommendation, and product recommendation), the advantage of POI recommendation is that POI recommendation is more closely connected to real life. POI recommendation requires users to visit a certain POI in the real world to generate check-in records. The user’s cost of contacting an item is low in product recommendation and music recommendation. However, in POI recommendation, there are high distance and time costs involved in the process of accessing a POI that make POI recommendation incomparable with other types of recommendations. These costs also affect whether a user will visit a certain POI.

In several previous studies, traditional user-based collaborative filtering, item-based collaborative filtering, latent factor models, and other algorithms based on matrix factorization and tensor factorization have been proven to be effective in many fields of recommendation [15–17]. In addition, many scholars have conducted research on neural network-based recommendation models and graph-based recommendation models [18–21]. However, for POI recommendations, the user behavior has an implicit feedback mechanism, which cannot directly obtain user preferences [22,23]. Therefore, the use of latent factor models to establish implicit features to connect users and POIs will have better applicability.

### 2.2 The influence of geographic factors

In most LBSNs, there is a function that accesses the location of a user, and this is what establishes the connection between the real world and cyberspace. In geography, many geographic analyses such as buffer analysis and window analysis are based on distance. Distance is an essential element in geography and an indispensable part of modern cartography. In addition, in the intersection of geography and data mining, Tobler’s first law of geography is the theoretical basis of spatial data mining. Tobler’s first law of geography states that "everything is related to everything else, but near things are more related to each other". Tobler’s first law of geography is the fundamental concepts of spatial autocorrelation. Spatial autocorrelation refers to the spatial dependence between objects in the same area, and is generally affected by inverse distance weighting.

Many scholars have studied the influence of geographic factors on POI recommendations [24–27]. Ye et al. [28] analyzed the spatial aggregation of user check-in behaviors and proposed a power-law relationship between the probability of user access and the distance. Yuan et al. [29] calculated the relationship between the probability of user access and the distance of multiple check-in records of multiple users and reached a conclusion similar to Ye’s. Cheng et al. [30] established a Gaussian model with multiple centers to analyze the influence of geographic factors and added the geographic influence to the MF model. Pan et al. [13] used the estimation of kernel density and the two-hop random walk approach to mine the geo-social relationships between users. The advantage of Pan’s method is that the kernel function has no-parameter estimation and can better simulate the distance distribution between POIs. In addition, there have been many studies that used geographic factors as the most significant factors that affect POI recommendations. They add geographic factors, time factors, social factors, popularity factors, comment information, and other contextual information into a joint framework to achieve higher performance and form better recommendations [6,31–33].

## 3. Proposed methods

GPMF is established based on the factorization model. Instead of modeling geographic factors by power law distribution or kernel density estimation, we attempt to model the geographic influence from the locational relationship between users, the distributional relationship between users and POIs, and the proximity and cluster relationship between POIs through geographic similarity, non-linear function, and KNN. Consequently, GPMF can more comprehensively exploit geographic information.

Due to the excellent performance of the latent factor model in POI recommendation, we used the MF model as the basis of our POI recommendation model. By decomposing the 0/1 check-in matrix ***R***_*m*×*n*_ (including *m* users and *n* POIs), the s-dimensional feature vectors of users and POIs can be obtained. Biased MF develops the basic matrix factorization by considering the biases [34] and has the better performance, so we adopt Biased MF as the basic form,

$${\hat{r}}_{ui}=b_{i}+b_{u}+p_{u}q_{i}^{T}$$ (1)

where ${\hat{r}}_{ui}$ is the performance of user *u* in POI *i*; *b*_*i*_ is the bias term of POI *i*; *b*_*u*_ is the bias term of user *u*; ***p***_*u*_ is the feature vector of user *u*; and $q_{i}^{T}$ is the transposition of the feature vector of POI *i*.

The objective function is shown in formula (2):

$$O=\min\underset{u,i}{\sum }w_{ui}{\left(r_{ui}-{\hat{r}}_{ui}\right)}^{2}+\lambda_{1}({\left\|p_{u}\right\|}_{F}^{2}+{\left\|q_{u}\right\|}_{F}^{2})+\lambda_{2}(b_{u}^{2}+b_{i}^{2})$$ (2)

where *w*_*ui*_ is the weight indicated by the visiting frequencies and defined by formula (3); a higher frequency indicates a large *w*_*ui*_; *r*_*u*i_ is used to mark whether user *u* has checked in POI *i*; *r*_*u*i_ = 1 if user *u* has checked in POI *i*, otherwise *r*_*u*i_ = 0; *λ*_1_ and *λ*_2_ are the parameters of the regular term. We used the stochastic gradient descent (SGD) to minimize the optimization function.

$$w_{ui}=\{\begin{array}{ccc} \tau F_{u,i}+1 & if & F_{u,i}>0\\ 0 & otherwise \end{array}$$ (3)

where *τF*_*u*,*i*_ is a monotonically increasing function with respect to the visit frequency *F*_*u*,*i*_. In this article, *τ* is taken as 0.1.

### 3.1 The influence of geographic factor among users

The user is the most important component and the main body of POI recommendation. From the perspective of user-based collaborative filtering, there is a relationship between users that is described by similarity. Users with high similarity are called similar users. Many studies have proven that similar users can be used to assist with recommendations [35].

Since people in the real world need to consider distance and time costs, the POI visited by people in the same area will have a higher degree of similarity. This is because many POIs are similar in function and user needs. Without considering the influence of other factors, people usually choose a POI with closer distance. Although the similarity of users in the user’s geographic space may not be as high as the top-n similar users obtained by a similarity calculation, the similarity of users in a shared geographic space has a higher interpretability in the real world. Therefore, after calculating the user similarity in the previous step, similar users in the geographic space of the user were selected based distance. Then, based on the preferences of the similar users, the influence of geographic factors on the relationships between users was added.

For the calculation of user similarity, we used the cosine similarity calculation method,

$$sim(u,v)=\frac{R_{u•}•R_{v•}}{\sqrt{R_{u•}}\sqrt{R_{v•}}}$$ (4)

where ***R***_*u*_ is the check-in status of user *u* at POIs in the 0/1 check-in matrix ***R***, and ***R***_*v*_ is the check-in status of user *v* at POIs.

To calculate the similarity of users in geographic space, we use the center of each user’s activity range as the calculation basis to find *t* geographically similar users (for example, the 10 closest neighbors) that are the geographically closest. The calculation method for the center of user activity range is as follows:

$$\bar{LON_{u}}=\frac{1}{\left|I_{u}\right|}\sum_{i\in I_{u}}LON_{i}$$ (5)

$$\bar{LAT_{u}}=\frac{1}{\left|I_{u}\right|}\sum_{i\in I_{u}}LAT_{i}$$ (6)

where *I*_*u*_ represents the set of all POIs that user *u* has checked in; *LON*_*i*_ represents the longitude of the POI *i*; *LAT*_*i*_ represents the latitude of the POI *i*; $\bar{LON_{u}}$ represents the longitude of user *u* in the center of the activity range; and $\bar{LAT_{u}}$ represents the latitude of user *u* in the center of the activity range.

### 3.2 The influence of geographic factor between users and POIs

Using a latent factor model in POI recommendation is important for connecting users with POI. Users and POIs are two types of objects in POI recommendation. The purpose of POI recommendation can be simply understood as recommending POI to users. The connection between users and POIs can affect POI recommendation, so the role of geographic factors in the relationship between users and POIs will be considered in this study. We analyze the influence of geographic factors in the user’s POI based on the life circle theory. The life circle is an activity area based on the temporal and spatial characteristics of human behavior and public resources [36]; it is the expansion of human life in space. It is worth mentioning that in this study we used the basic life circle, that is, the living space that meets people’s daily needs (shopping, medical treatment, dining and other public service facilities).

Although many researchers have analyzed the check-in data and found that there is a power-law relationship between the user’s check-in probability and the distance, whether this check-in probability is consistent with the real word remains to be further verified for the power law formula considering geographic factors. The results of Ye et al. [28] and Zhang et al. [8] suggest that most check-in records (two check-ins of the same user) are generated at distances above 100km. The data they analyzed included Foursquare datasets, Whrrl datasets, Yelp datasets, and Breadtrip datasets. However, the power law relationship is more obvious in the range when the check-in records are sparse and the distance is less than 100km, while the power law relationship is not obvious in the range when the check-in records are dense and the distance is more than 100km. For example, Singapore is a country with a land area of ​​728.3 square kilometers. The land area spans about 55km from east to west and 27km from north to south. Considering the POI recommendation, most visits of ordinary urban residents should still be in this city. Although these residents may visit other cities that are farther away, it will not be the main part of their visit. In addition, there are already thousands of POIs in a city, making it difficult to complete higher-performance POI recommendations. The challenge of POI recommendations will increase even further if we consider POIs outside the city. Therefore, based on the theory of life circle, which has behavioral geography activity analysis as its core, this study considered the geographic influence from a more practical perspective.

The main steps of considering the influence of geographic factor between users and POIs were as follows. (1) We added a life circle on the LBSN and selected the radius of the life circle according to the actual situation and research of relevant behavioral geography. (2) We calculated the activity center on the user’s historical check-in data. (3) According to the user’s activity center and life circle, all POIs were classified as either being inside or outside of the life circle. Using the life circle to consider the influence of geographic factors, it is possible to utilize local information to analyze geographic influences in a small area instead of analyzing the overall situation. The life circle allows for a more fine-grained simulation of real-world behavior in geographical space; such a simulation would better model the actual user situations. Assuming that the user check-in behavior is random, the user check-in data are discrete points on the user’s activity track. The user check-in data and user activity track data obey the same distribution. Hence, the user’s life circle and activity center can be calculated with greater accuracy.

To meet the above calculations, we used a nonlinear function to simulate the influence of geographic factors between users and POIs, with consideration of the life circle theory,

$$\{\begin{array}{c} D_{ui}=1,\;if\;dis(i|u)\leq d\\ D_{ui}=0,\;if\;dis(i|u)>d \end{array}$$ (7)

where *D*_*ui*_ = 1 means that POI *i* is in user *u*’s life circle; *D*_*ui*_ = 0 means that POI *i* is not in user *u*’s life circle; *dis*(*i*|*u*) is the distance between POI *i* and the center of user *u*’s activity range; and *d* is the radius of the life circle obtained based on the life circle theory and experimental test. It is worth noting that *d* may be different in different places.

### 3.3 The influence of geographic factor among POIs

The most common analysis in geography is the analysis of the relationship between objects. For example, POI is common in geographical analysis. The distance factor of geospatial analysis is the most basic element of analyzing the relationship between POIs. We used KNN to calculate the geographic neighbors of the POIs, and used the visited frequency of POIs to measure the impact between POIs. We included geographic factors other than the distance when considering the role of geographic factors between POIs; these factors were often ignored in previous studies. The first is the aggregation information of the POI; this information is calculated according to the geographical coordinates of the POI to obtain the area where multiple POIs are clustered. It should be noted that the calculation method used in this study is point density rather than kernel density. This is because the longitude and latitude of the POI were regarded as point coordinates; the weights of the points in the same search area were the same when density analysis was performed; and the weights should not change with the distance from the search center. The aggregation information of the POI can be used to construct the POI cluster and thus assist in the subsequent POI recommendation task. Taking into account Tobler’s first law of geography, this study considered some attributes of POI (such as functions, consumption levels, design concepts, and Levels) to be similar in the same cluster. In the real world, Huaqiangbei in Shenzhen and Akihabara in Japan are more obvious. The transaction share in the Huaqiangbei area is mainly electronic product transactions. Most of the regional industries are related to electronic products. Akihabara is also based on the sale of electronic digital products, and it is also a mecca for ACGN (Anime, Comic, Game, Novel) enthusiasts. Most of the POI functions here are related to electronic digital products and ACGN. Therefore, clustering POIs into clusters according to POI positions and enhancing the correlation between POIs in the same cluster can provide more recommended information for POIs in the same cluster. In addition, most studies explored the effect of distance when considering the impact of geographic factors on POI. However, these studies analyzed the distance relationship between two POIs or the transition probability between POIs. We not only considered the distance factor but also the regional influence of geographic factors on POI recommendations. The main features of our study are (1) the calculation of multiple POI clusters based on the POI density and the subsequent use of the cluster’s influence to assist with recommendations, and (2) the further consideration of the circulation of POIs belonging to the constructed POI cluster. Inspired by the temporal-spatial proximity proposed by Li et al [37] who stated that flow is the key to influencing proximity, we used regional average popularity as the criterion for judging flow. The POI with high popularity attracts more traffic, and the POI with low popularity attracts less traffic. For a POI cluster, we suppose that users tend to shift from POIs with low popularity to POIs with high popularity, and there are exclusive circulation channels between POIs in the cluster.

Relative to the whole city, POI clusters can analyze the role of geographic factors on a more fine-grained level. Secondly, according to the POI cluster, the roles of geographic factors were divided into inter-cluster geographic influence and intra-cluster geographic influence. For the inter-cluster geographic influence, the average POI popularity in the cluster was taken to be the influence of the POI cluster, and then the influences of all POI clusters were linearly normalized to obtain the normalized POI cluster influence. For the intra-cluster geographic impact, the median of the number of POI visits in the cluster was used as the standard to normalize the popularity of each POI in the cluster. POIs with a normalized popularity greater than 0 are the POIs that attract users; POIs with a normalized popularity of less than 0 are the ones that lose users. The closer the value is to 1 or -1, the stronger the degree of user attraction. For a POI cluster, all POIs in the cluster constitute the influence of the cluster together; there is still a popularity gap and competition between POIs in the same cluster. Therefore, we used POI normalized popularity to measure whether a POI attracts or loses users. By classifying geographic influence into inter-cluster geographic influence and intra-cluster geographic influence, the cooperation and competition relationship between POIs in the real world can be better simulated with better interpretability.

### 3.4 Unified model construction

From the most primitive MF model, the role of geographic factors was gradually added, and the influence of geographic factors among users was integrated,

$${\hat{r}}_{ui}=b_{i}+b_{u}+(p_{u}+\alpha_{E}\sum_{u_{E}\in U_{u}^{E}}sim(u,u_{E})p_{u_{E}}+\alpha_{G}\sum_{u_{G}\in U_{u}^{G}}\frac{1}{dis^{c}(u,u_{G})+1}p_{u_{G}})q_{i}^{T}$$ (8)

where *α*_*E*_ and *α*_*G*_ are the influence coefficients of similar users calculated using the user’s explicit attributes and geographic similarity, respectively; $U_{u}^{E}$ is the set of *t* most similar users found by using the 0/1 check-in data according to the similarity calculated by formula (4); $U_{u}^{G}$ is the set of *t* most similar users calculated using user geographic similarity; *sim*(*u*,*u*_*E*_) is the similarity between user *u* and user *u*_*E*_; and *dis*^*c*^(*u*,*u*_*G*_) is the distance between the activity center of user *u* and user *u*_*G*_.

Secondly, we integrate the influence of geographic factors between users and POIs,

$${\hat{r}}_{ui}=b_{i}+b_{u}+(p_{u}+\Delta_{E}+\Delta_{G})(q_{i}^{T}+\alpha_{L}\sum_{i_{L}\in I_{u,d}^{L}}\omega q_{i_{L}}^{T})$$ (9)

$$\Delta_{E}=\alpha_{E}\sum_{u_{E}\in U_{u}^{E}}sim(u,u_{E})p_{u_{E}}$$ (10)

$$\Delta_{G}=\alpha_{G}\sum_{u_{G}\in U_{u}^{G}}\frac{1}{dis^{c}(u,u_{G})+1}p_{u_{G}}$$ (11)

where *α*_*L*_ is the coefficient that controls the influence of geographic factors in combination with the theory of the life circle; $I_{u,d}^{L}$ is the POI set that is less than *d*km from the activity center of the user *u*; and *ω* is the reciprocal of the length of the set $I_{u,d}^{L}$.

Finally, we integrate the influence of geographic factors among POIs,

$${\hat{r}}_{ui}=b_{i}+b_{u}+(p_{u}+\Delta_{E}+\Delta_{G})(q_{i}^{T}+\Delta_{L}+\alpha_{D}\sum_{i_{D}\in I_{i}^{D(K)}}F_{i_{D}}q_{i_{D}}^{T})+\alpha_{\mathrm{int}er}C_{c(i)}^{\mathrm{int}er}+\alpha_{\mathrm{int}ra}C_{i}^{\mathrm{int}ra}$$ (12)

$$\Delta_{L}=\alpha_{L}\sum_{i_{L}\in I_{u,d}^{D}}\omega q_{i_{L}}^{T}$$ (13)

$$C_{c(i)}^{inter}=f_{1}(\frac{1}{\left|c\right|}\sum_{i\in c}F_{i})$$ (14)

$$C_{i}^{intra}=f_{2}(F_{i})$$ (15)

where *α*_*D*_ is the influence coefficient of the distance between POIs; $I_{i}^{D(K)}$ is the set of geographic neighbors determined by the KNN algorithm for POI *I*; $F_{i_{D}}$ is the number of visits of POI *i*_*D*_; *α*_*inter*_ and *α*_*intra*_ are the inter-cluster influence coefficient and the intra-cluster influence coefficient, respectively; $C_{c(i)}^{inter}$ is the influence of cluster c to which POI *i* belongs; $C_{i}^{intra}$ is the influence of POI *i* in the cluster; *f*_1_ and *f*_2_ are normalization functions; *f*_1_ is linear normalization; *f*_2_ is normalization using median; and *F*_i_ is the number of times that POI *i* has been visited.

### 3.5 Optimization

We utilized SGD, which is commonly used in the field of machine learning, to update the parameter:

$$\theta \leftarrow \theta -eta\frac{\partial O}{\partial \theta }$$ (16)

where *θ* represents the parameter that needs to be updated, and *eta* is the learning rate.

## 4. Experiment or experiments

We performed a POI recommendation experiment with a Geographic Personal Matrix Factorization model (GPMF) and compared the performance with the baseline methods.

### 4.1 Experimental settings

The real-life check-in dataset used in this experiment is the Foursquare dataset provided by Yuan et al. [29]. The dataset includes 2 321 users, 5 596 POIs, and a total of 194 108 check-in records; the data density is 0.81%. Each check-in record in the dataset is generated by a user with a unique identifier accessing a POI with the unique identifier, and the check-in location (latitude and longitude format) and check-in time are recorded. The experimental dataset was divided into two parts, the first 80% was used as the training set, the last 20% was used as the testing set. All latent features were calculated by formula (12) and formula (16), and then *k* POIs were recommended for each user according to the calculated predicted value.

### 4.2 Evaluation metrics

The evaluation metrics depend on the recommendation task [38]. The task of the proposed model is to recommend top-n recommendations to users. Therefore, we used two indicators to evaluate the performance of the proposed model: *Precision@k* and *Recall@k*. The *Precision@k* refers to the ratio of the recommended correct POI number to the recommended number *k*, and the *Recall@k* refers to the ratio of the recommended correct POI number to the number of POIs actually visited by the user in the test set. Formally, the metrics are formulated as follows:

$$Precision@k=\frac{1}{\left|U\right|}\overset{\left|U\right|}{\underset{u=1}{\sum }}\frac{S_{u}\cap M_{u}(k)}{k}$$ (17)

$$Recall@k=\frac{1}{\left|U\right|}\overset{\left|U\right|}{\underset{u=1}{\sum }}\frac{S_{u}\cap M_{u}(k)}{\left|S_{u}\right|}$$ (18)

where *S*_*u*_ is the set of POIs that user *u* has visited in the testing set but has not visited in the training set, and *M*_*u*_(*k*) is the set of *k* POIs recommended by the GPMF.

### 4.3 Baseline methods

The proposed method is compared to the other four baseline methods on the Foursquare dataset. The four methods are:

1. MF-0/1: A method that uses 0–1 check-in matrix ***R*** to perform matrix factorization, where if user *u* has a check-in record at POI *i*, ***R***_*ui*_ is 1; otherwise, it is 0.
2. MF-Frequency: A method of matrix factorization using the check-in frequency matrix.
3. WMF: A method that can effectively solve the implicit feedback. It improves the recommendation performance by adding a weight matrix to the matrix factorization [34].
4. Geo-MF: A POI recommendation algorithm that combines geographic influence and matrix factorization [39].

### 4.4 Result and analysis

#### 4.4.1 Parameter tuning

In GPMF, there are many parameters that need to be adjusted, including parameters *α*_*E*_, *α*_*G*_, *α*_*L*_, *α*_*D*_, *α*_*inter*_, *α*_*intra*_, *K*_*E*_, *K*_*G*_, *K*_*D*_, and *d*. Other parameters were predefined; the learning rate *eta* was set at 0.001; the regularization parameter *λ* was set at 0.00001; and the dimensions of latent factors were set to 15. We used the grid search to adjust the parameters to the optimal combination and obtain the best performance.

After the adjustments, we set the parameter *α*_*E*_ to 1.2, *α*_*G*_ to 0.00001, *α*_*L*_ to 0.001, *α*_*D*_ to 0.000001, *α*_*inter*_ to 0.0001, *α*_*intra*_ to 0.001, *K*_*E*_ to 15, *K*_*G*_ to 10, *K*_*D*_ to 10, and *d* to 1.5km. The experimental adjustment process of each parameter is shown in Figs 1–10.

**Fig 1:**
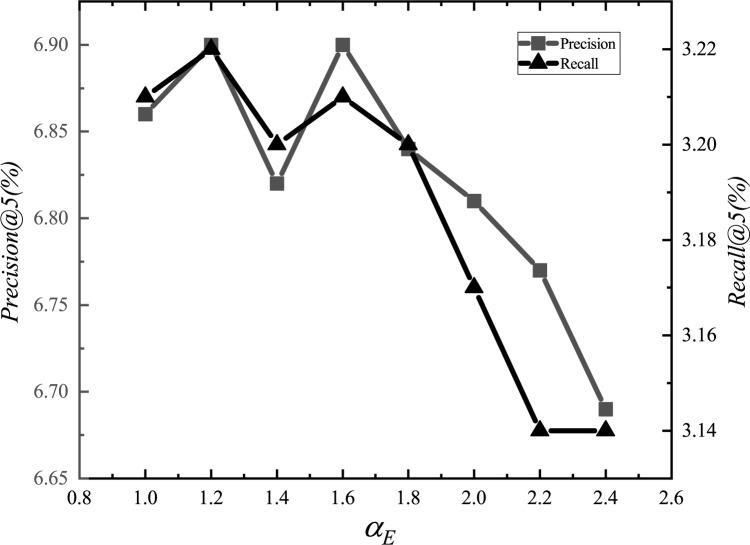
Tuning parameters(*α*_*E*_).

**Fig 2:**
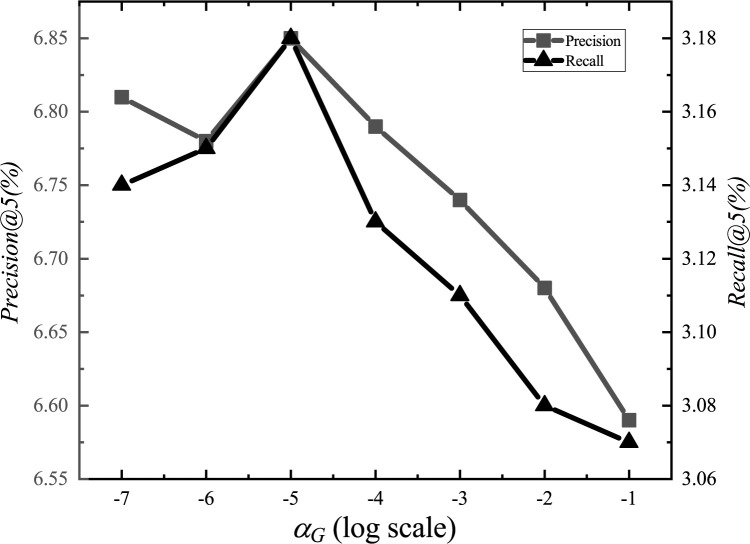
Tuning parameters(*α*_*G*_).

**Fig 3:**
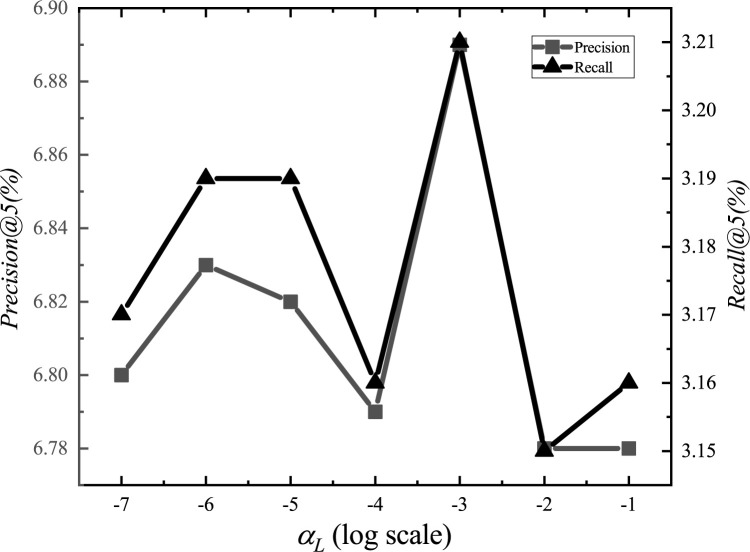
Tuning parameters(*α*_*L*_).

**Fig 4:**
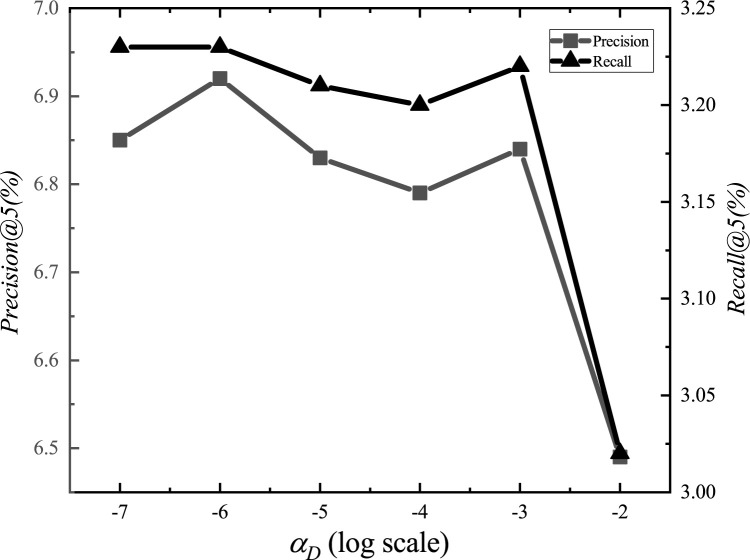
Tuning parameters(*α*_*D*_).

**Fig 5:**
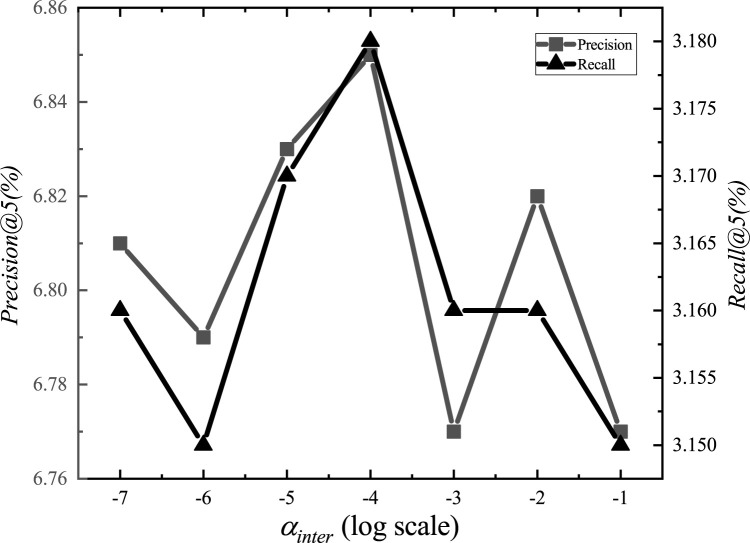
Tuning parameters(*α*_*inter*_).

**Fig 6:**
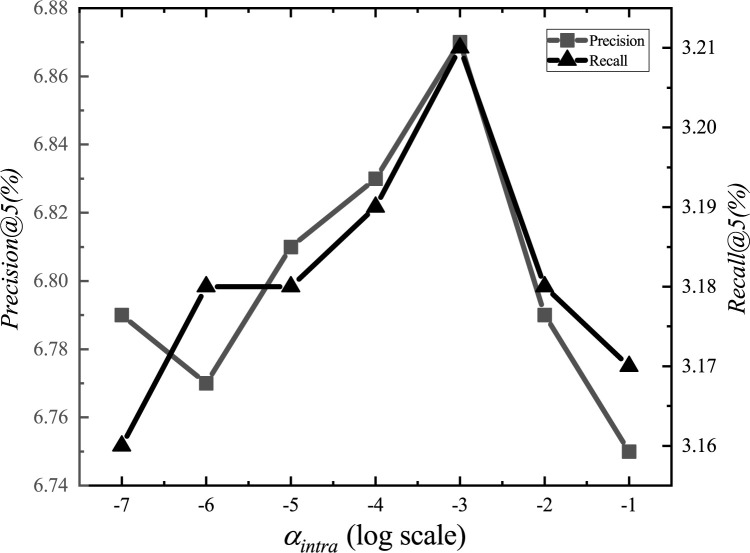
Tuning parameters(*α*_*intra*_).

**Fig 7:**
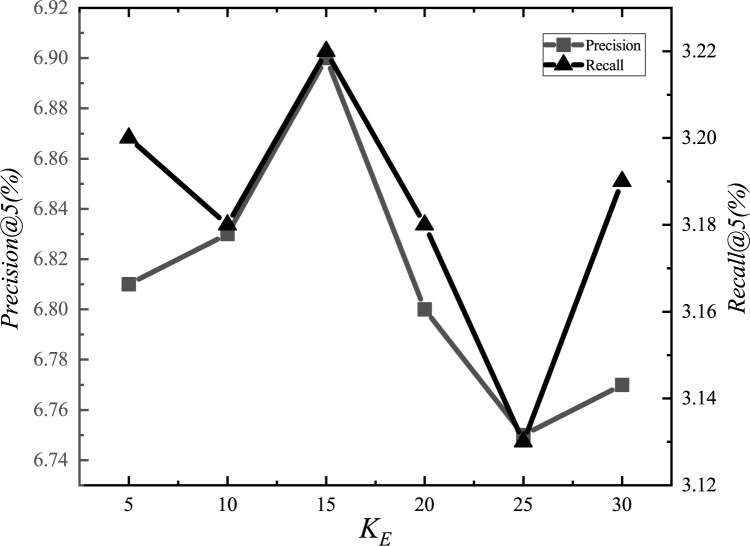
Tuning parameters(*K*_*E*_).

**Fig 8:**
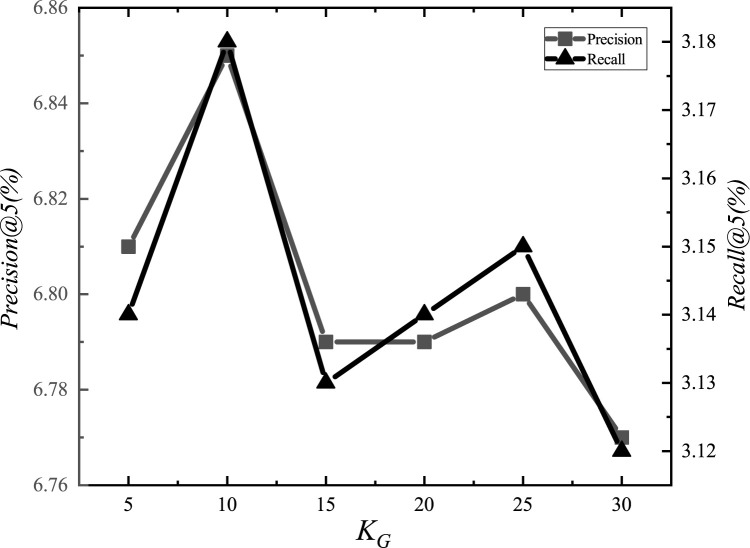
Tuning parameters(*K*_*G*_).

**Fig 9:**
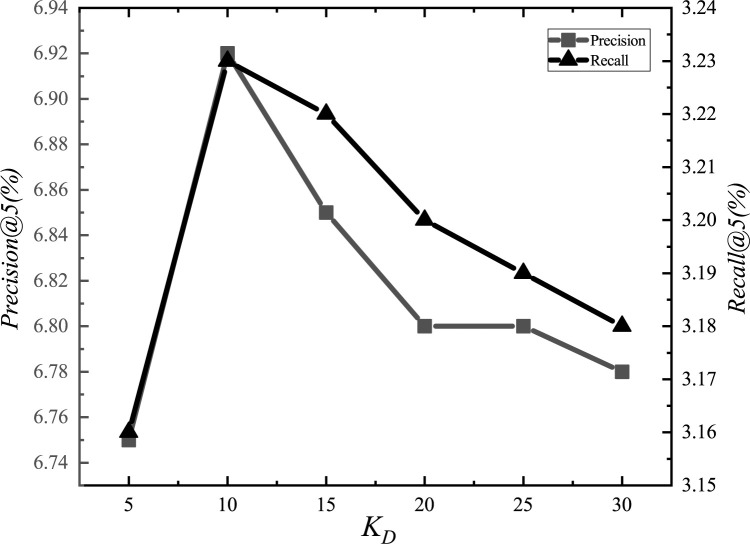
Tuning parameters(*K*_*D*_).

**Fig 10:**
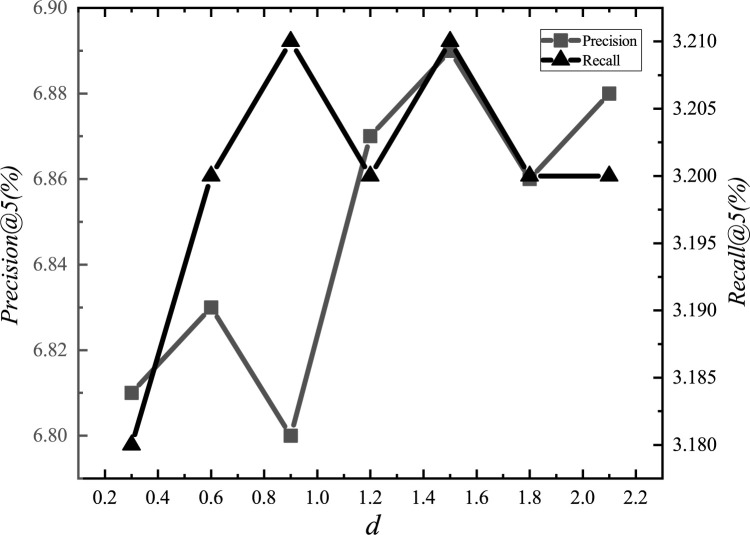
Tuning parameters(*d*).

The parameter adjustment results show that all parameters have an impact on the performance of the model. Among them, the most influential parameter is *α*_*D*_, which controls the distance between POIs.

#### 4.4.2 Comparison to baselines

**Fig 11:**
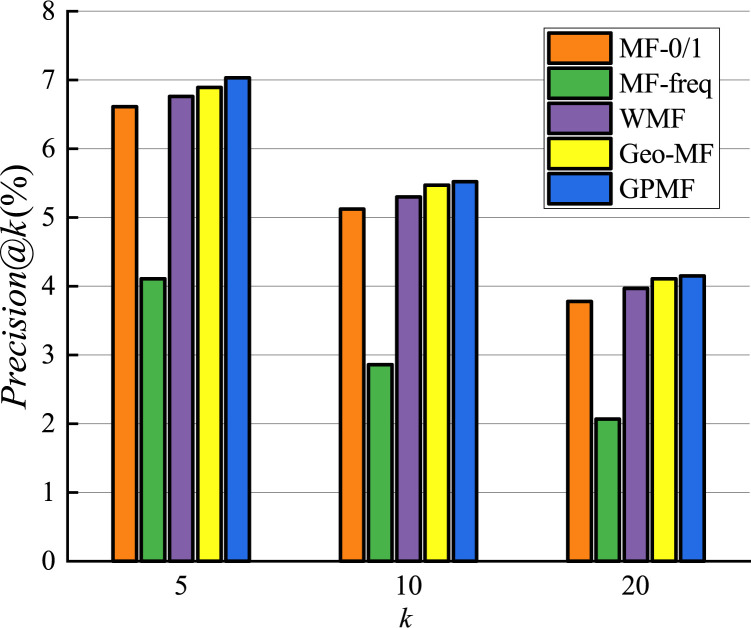
Recommended precision comparison.

**Fig 12:**
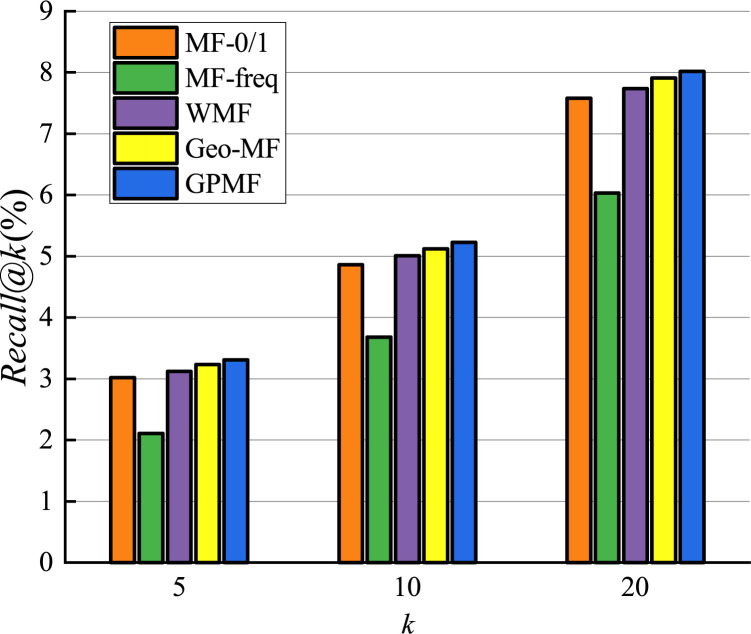
Recommended recall comparison.

We experimentally investigated the results of comparison between the proposed GPMF model and other baseline methods. The results on the Foursquare dataset are shown in Figs 11 and 12.

Since the model is a recommendation list sorted by scores, when the number of recommended POIs is 5, the result obtained by the recommendation algorithm is of highest importance [40]. The performance of MF-freq is the worst in terms of precision and recall among all baseline methods. This is because MF-freq is directly calculated by the user’s check-in frequency, and the gap between the user’s check-in frequency is very large. Active users can check in hundreds or even thousands of times in the Foursquare dataset, while inactive users often check in only a few times. This gap in the number of check-ins makes it difficult for the MF-freq model to accurately quantify the preferences of different users. Therefore, the recommended performance is the worst. The recommended performance of MF-0/1 is better than that of MF-freq, but MF-0/1 does not consider the impact of user check-in frequency, while WMF considers the impact of user check-in frequency and uses a weighted indirect method rather than directly use check-in frequency.WMF reduces the impact of the large gap in check-in frequency, so the performance of WMF has been further improved. The performance of Geo-MF is better than that of WMF. This is primarily because Geo-MF considers the using weighting to alleviate the implicit feedback problem and uses geographic information to assist with recommendations. When the number of recommendations is 5, the performance of GPMF is better than Geo-MF in terms of recommended precision and recall by 2.0% and 2.5%, respectively. This is because GPMF considers the locational relationship between users, the distributional relationship between users and POI, and the proximity and clustering relationships between POIs. Therefore, GPMF has a more comprehensively considers the influence of geographic factors and specific modeling of the influence of geographic factors between different objects. The good recommendation performance of GPMF shows that comprehensive consideration of the role of geographic factors in POI recommendation can better improve recommendation efficiency.

#### 4.4.3 Effect of the geographic factors from different perspectives

To better understand the specific effects of geographic factors between users, between users and POI, and between POIs, we investigated the impact of geographic factors on the performance of POI recommendations from different perspectives. First, we expanded the GPMF including GPMF-E, which considers the user similarity; GPMF-G, which considers the geographic factors between users; GPMF-L, which considers geographical factors between users and POIs; and GPMF-D, GPMF-inter, and GPMF-intra, all of which consider geographical factors between POIs. Moreover, GPMF-D considers the proximity relationship between POI and POI; GPMF-inter considers the relationship between clusters; and GPMF-intra considers the relationship within clusters.

**Fig 13:**
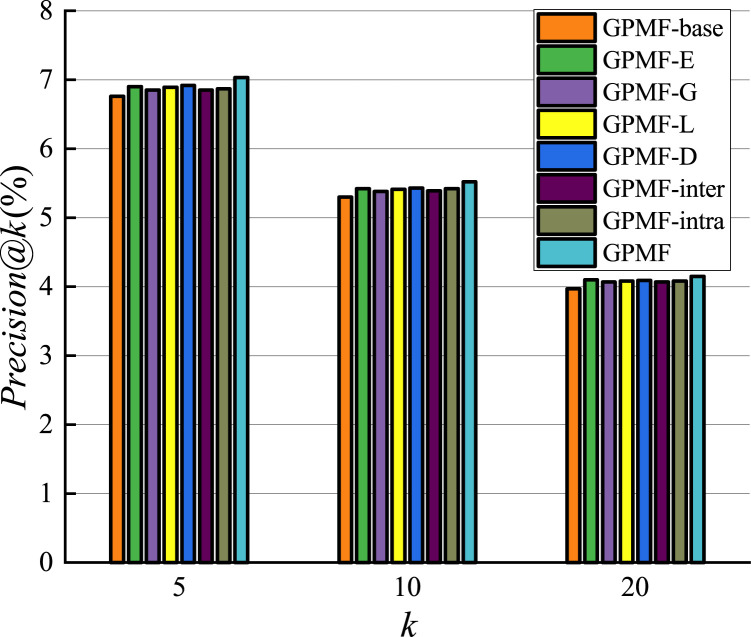
Comparison of the effects of different factors(precision).

**Fig 14:**
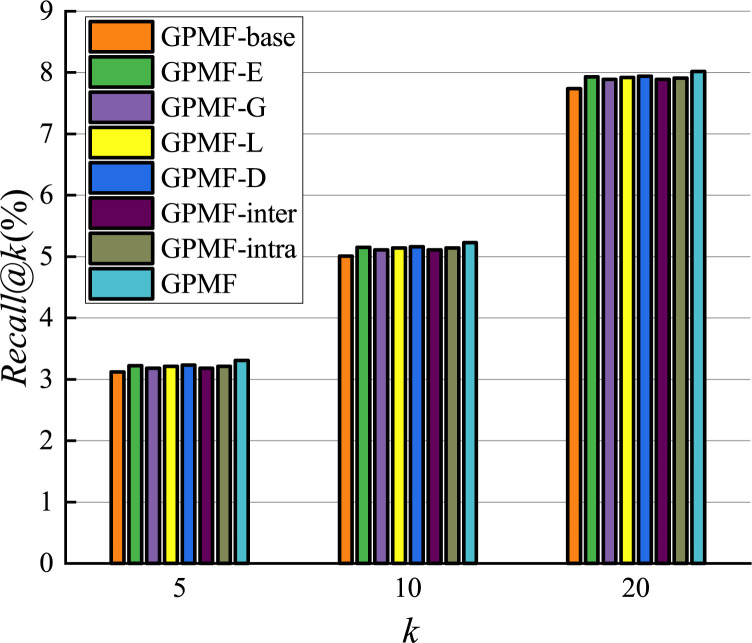
Comparison of the effects of different factors(recall).

After setting up various parameters through experiments on the Foursquare dataset, the results corresponding to different versions of the GPMF model are shown in Figs 13 and 14. Comparing the precision and recall of the different GPMG models, it can be concluded that the recommendation system performance can be improved by considering the role of geographic factors from the three perspectives of users-users, users-POIs, and POIs-POIs. When the number of recommendations was 5, relative to the GPMF-base that does not consider any additional information, the precision of GPMF-G, GPMF-L, GPMF-D, GPMF-inter, GPMF-intra, and GPMF were increased by 1.3% and 1.9%, 2.4%, 1.3%, 1.6% and 4.0%, respectively; and the corresponding recalls were increased by 1.9%, 2.9%, 3.5%, 1.8%, 2.9% and 6.1%, respectively. For GPMF-E, the recommended precision and recall were improved by 2.1% and 3.2%, respectively. The recommendation performance was improved by the construction of the GPMF-E method due to the combination of user-based collaborative filtering and latent factor model, which are two different recommendation methods. The GPMF-D method demonstrated the highest precision and recall of recommendation results. The proximity relationship between POIs can best improve the performance of recommendation model, so the recommendation model incorporating geographic information should focus on the impact of the proximity relationship between POIs. When the recommended number is 10 or 20, the results are similar and will not be repeated.

## 5. Discussion and conclusions

In view of the lack of effective use and representation of geographic information in current research, this paper proposes the POI recommendation model GPMF. We considered the impact of geographic information from the perspective of the relationships between different objects. Specifically, the relationships are divided into the locational relationship between users and users, the distributional relationship between users and POI, and the proximity and clustering relationships between POIs. These relationships are then integrated into the MF model. By subdividing the impact of geographic information on different objects, the role of geographic information can be more effectively simulated, with better interpretability and expansibility. Experiments on the Foursquare check-in dataset revealed that (1) the performance of GPMF is better than the current commonly used POI recommendation algorithm, and (2) the performance of the recommendation algorithm can be improved more effectively by describing geographic information through proximity relations.

Compared to other types of geographic information, the proximity information has a higher performance improvement for POI recommendation. However, the joint improvement effect of different types of geographic information on the recommendation performance did not achieve a superimposed effect, confirming the existence of joint promotion and overlapping functions. Moreover, the combination of user-based collaborative filtering and latent factor model can help improve the performance of POI recommendation. The use of geographic information can help improve POI recommendation performance. However, the use of geographic information by GPMF may not achieve the best results. Both deep learning and graph-based methods demonstrate excellent performance, so we will explore these methods further as we continue investigating POI recommendation combined with geographic information. Since many commonly used methods utilize time information, social information, and other contextual information in POI recommendations to improve performance, we will incorporate time and social relationships and categories into the GPMF model to study the influence modes of these information.

## Supporting information

S1 File The data used in the experiment is in poidata. (ZIP)
